# Antimutagenic, antitumor and estrogen receptor binding activity of the rare plant *Shortia galacifolia*: An ethnobotanical and chemosystematic approach

**Published:** 2019

**Authors:** Sandra L. Gray, Brett R. Lackey, Patricia L. Tate

**Affiliations:** 1 *Endocrine Physiology Laboratory, Animal & Veterinary Science Department, Clemson University, Clemson, South Carolina, USA*; 2 *School of Nursing, Clemson University, Clemson, SC *; 3 *Seneca Creek Organics, Seneca, SC*

**Keywords:** Antimutagenic, Antitumor, Bioassay, Estrogen receptor, Ethnopharmacology

## Abstract

**Objective:**

*Shortia *and other members of the Diapensiaceae family have ethnomedicinal history in both Eastern and Western hemispheres. Based on ethnopharmacological and chemosystematic evidence, pharmacological and toxicological bioassays were conducted on the rare plant Oconee Bell,* Shortia galacifolia*.

**Materials and Methods:**

Extracts were examined in assays for antimutagenicity, antitumor and estrogen receptor (ER)-binding activity. Antitumor activity was assessed by the tumor induction assay (TiA), using *Agrobacterium tumefaciens* based on its ability to transform plant tissue. Antimutagenicity was examined using the Ames bacterial reverse mutation test. Recombinant human ERα and ERβ proteins were utilized to screen extracts for receptor selectivity.

**Results:**

All concentrations of extracts inhibited *A. tumefaciens*-induced tumor formation on potato discs, with the mature rhizome extracts having the most marked inhibition. All three plant extracts significantly inhibited the formation of histidine-independent revertant colonies after exposure to the mutagen 2-aminoanthracene (2-AA) in the Ames *Salmonella *mutagenicity assay. In the ER binding assays, ERβ, but not ERα, displayed affinity for *Shortia* extracts.

**Conclusion:**

Antitumor, ER binding and antimutagenic activities of *S. galacifolia* extracts were identified using rapid bench-top assays and warrant further investigations.

## Introduction

What is the best approach to screening for phytomedicinals when ethnomedicinal knowledge is lacking? In such cases, an approach involving chemosystematics, also called pharmaphylogeny, may be useful (Cozzo, 2004 ▶; Pieroni and Vandebroek, 2007 ▶; Vick, 2011 ▶). Chemosystematics refers to a screening approach that focuses on specific chemicals, classes of chemicals or bioactivity within specific plant taxa (Atanasov et al., 2015 ▶; Pieroni and Vandebroek, 2007 ▶; Song et al., 2016 ▶).

**Figure 1:**
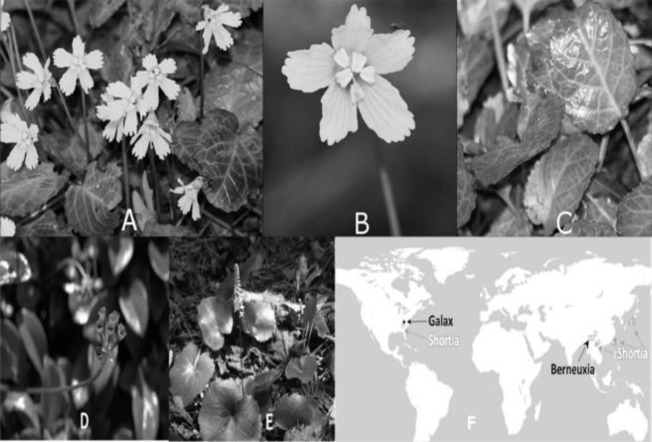
*Shortia galacifolia*, the Oconee Bell, (A) Plants growing on the forest floor near the Jocassee Valley, SC. (B) Close-up of the flower. (C) Leaves in winter, compared with (D) *Berneuxia* (Averater, 2017 ▶) and (E) *Galax* (Bodner, 2017 ▶). (F) Distribution of three members of Diapensiaceae including *Shortia, Galax and Berneuxia*. *Shortia* species include *S. galacifolia* in Eastern North America as well as *S. sinensis*, *S. rotundifolia*, *S. uniflora* and S. *soldanelloides *in Asia (GBIF, 2017 ▶)

 The Diapensiaceae is one such family that may benefit from this approach and includes five genera: *Shortia*, *Galax* and *Berneuxia*, shown in Figure 1, *Pyxidanthera* and *Diapensia* (Ronblom and Anderberg, 2002 ▶). The family has a disjunct distribution, described in Figure 1, with members occurring in eastern North America and the Sino-Japanese Floristic Region (Ronblom and Anderberg, 2002 ▶). In the following paragraphs, we will present a chemosystematic rationale for examining *Shortia galacifolia* T. & G. for potential medicinal activity (see Table 1). 

 The plant that is the focus of this study, *Shortia galacifolia*, is indigenous to tiny isolated areas of the Southern Appalachian Mountains of North Carolina and the Jocassee Gorges of upstate South Carolina (Davies, 1955 ▶; Dunn and Jones, 1979 ▶). It is a rhizomatous, low-growing, evergreen plant with dark, glossy green leaves that turn reddish in the winter (Davies, 1955 ▶; Dunn and Jones, 1979 ▶). It blooms in early spring with a single bell-shaped white or pink flower on a slender peduncle 6-8 inches high as shown in Figure 1, giving rise to its common name, the Oconee Bell. This rare plant was collected for scientific identification in 1787 by André Michaux and later encountered in 1839 by Asa Gray in a Paris herbarium (Jenkins, 1946 ▶; Vivian, 1967 ▶). 

 A bit of ethnobotanical evidence was provided in Michaux’s notes: that the Cherokee people inhabiting the Keowee area at the time Michaux collected his *Shortia* specimen were familiar with the plant and noted that it had a good taste and pleasing aroma (Jenkins, 1946 ▶; Vivian, 1967 ▶). Further ethnobotanical information on *S. galacifolia* has been lacking. Transmission of the ethnobotanical knowledge of this plant, like that of its related plant *Galax,* may have been negatively impacted by several factors including decades of warfare in the Keowee area before the development of the Cherokee writing system, the forced relocation of the Cherokee during the Indian Removal and the subsequent inundation of this historically important area in the creation of Lake Keowee (Cozzo, 2004 ▶; Pieroni and Vandebroek, 2007 ▶; Vick, 2011 ▶). 

 However, there is ethnomedicinal evidence for a different *Shortia* species from China, *Shortia thibetica* Decne. Franch is synonymous with *Berneuxia thibetica* Decne and *Berneuxia yunnanensis* H.L.Li, 岩筋菜, Yán Jīn Cài (shown in Figure 1; to avoid confusion we will use *B. thibetica* designation for the remainder of this article). *B. thibetica* is endemic to the southwest of China, including the Yunnan province, and is used in Traditional Chinese Medicine (TCM) as a cure for asthma, overstrain and cough (Wang et al., 1998 ▶). This work represents the first research, to our knowledge, of *S. galacifolia *in pharmacological and toxicological assays. Bioassays can provide a useful methodology for examination of plant extracts for antitumor, mutagenic and estrogen receptor (ER)-binding activity. The tumor induction assay (TiA) is based on the unique *Agrobacterium tumefaciens* characteristic of inserting a portion of its tumor-inducing (Ti) plasmid into wounded plants resulting in tumor-like growth, commonly known as Crown Gall disease (Galsky et al., 1981 ▶). Potential antitumor activity of plant extracts can be evaluated by measuring inhibition or stimulation of *A. tumefaciens-*induced tumor formation on potato discs treated with the plant extract (Galsky et al., 1981 ▶). Activity in the TiA has been correlated with antitumor activity in mammals (McLaughlin et al., 1998 ▶). 

 The Ames *Salmonella* mutagenic assay is a bacterial reverse mutation test which uses a histidine-dependent auxotrophic mutant of *Salmonella typhimurium* that lacks normal DNA repair mechanisms and cannot grow on histidine-free media (Mortelmans and Zeiger, 2000 ▶). Mutagenic changes are assessed by the number of colonies. Conversely, the degree to which a plant extract inhibits diagnostic mutagens can be used to evaluate antimutagenic and possibly anticarcinogenic potential (Friedman and Smith, 1984 ▶). 

 Chemosystematic analysis indicates that several members of Diapensiaceae display activities that may result from targeting the estrogen receptors (Garrett, 2003 ▶; Wang et al., 1998 ▶). ERα and ERβ can be utilized as a screening tool for extracts or individual chemicals with selective ER receptor modulatory (SERM) bioactivity without cell culture and eliminating any potential for crosstalk (Gray et al., 2004 ▶; Lackey et al., 2001 ▶). The objective of this study was to use these bench-top assays to examine *S. galacifolia* for antitumor, estrogen-receptor modulating and antimutagenic characteristics.

## Materials and Methods

**Plant material** **s**

*Shortia *specimens (leaves and rhizome) were collected with permission from native populations in Oconee County (34.9516° N, 82.9463° W), South Carolina and from a transplanted population at the South Carolina Botanical Garden in Clemson, South Carolina. Species identification was verified by Patrick D. McMillian, Director of the South Carolina Botanical Garden (Clemson University Herbarium Identification #63415) and by David Bradshaw, Professor of Horticulture, Clemson University. *S. galacifolia* Torr. & A. Gray is an accepted name and was checked with http://www.theplantlist.org on 2/4/2018. Oconee bell and Acony bell are two common names.

Red raspberry leaf (RRL; *Rubus idaeus*) extracts were a generous gift from Dr. J. L. McLaughlin (Nature’s Sunshine Products Inc., Spanish Fork, Utah). Ethyl acetate extracts of RRL, 10g sonicated in 100 ml for 2× 2 hr, were evaporated under N_2 _to a volume and shipped to the Endocrine Physiology Laboratory at Clemson University, Clemson, SC for analysis. 

**Extraction**

*Shortia* samples were washed under running water for 5 min and allowed to air-dry at room temperature overnight. Plants were divided into leaf material, new growth rhizome and mature rhizome and dried in an oven at 40^o ^C for 48 hr. Samples (5 g) were ground, extracted with 80% methanol, placed on a lateral shaker and mixed for 8 hr at room temperature (further details are provided in the Supplementary material). Samples were centrifuged and the pellet was re-extracted, evaporated to dryness, reconstituted with 100% ethanol or DMSO (1g/mL), filtered and stored in the dark at 25^o ^C.

Dried RRL extracts, 2 g equivalent, were suspended overnight in 3 mL hexane. Samples were then extracted with 3 mL of 80% methanol, and centrifuged at 1800 g for 15 min. The hexane supernatant was removed and extracted again with 80% methanol. Methanolic fractions were combined, filtered with 0.45 μm Acrodisc® and evaporated to dryness on a heated DriBath (45^o ^C) under a gentle stream of filtered air. Extracts were reconstituted to a concentration of 1g original RRL /ml 100% ethanol. 

**Antitumor assay**

Antitumor activity of *Shortia* extracts was examined by using the TiA method detailed by McLaughlin’s laboratory (McLaughlin et al., 1998 ▶). Preliminary experiments on hexane and methanol solvent suitability with *Agrobacterium* tumor induction experiment, were conducted (data not shown). Samples were reconstituted and diluted in DMSO for assay testing. Further details on the methodology are presented in the Supplementary material.

*Shortia* extracts (1 g/ml) were diluted in 50% ethanol to obtain four concentrations: 10 mg/ml (1:100), 1 mg/ml (1:1000), 0.1 mg/ml (1:10,000), and 0.01 mg/ml (1:100,000). Each tissue and dilution was tested by use of two separate culture dishes with twelve potato discs each for a total of 24 replicates per treatment. Three controls were used: (1) positive *A. tumefaciens* control in which no test sample was added, (2) a positive inhibitory control in which camptothecin was added and (3) a solvent control which contained 50% ethanol but no *A tumefaciens*. Discs contaminated with observable bacterial or fungal growth were not counted. Experiments were repeated three times and data were analyzed for statistical differences by using analysis of variance (ANOVA, SAS Institute; Cary, NC).

**Antimutagenic assay**

Antimutagenic characteristics of leaf, new growth rhizome and mature rhizome extracts were determined by using the Ames *Salmonella/*microsome mutagenic test with modifications (Mortelmans and Zeiger, 2000 ▶). Extracted plant samples were diluted 1:1000 in DMSO.

**Figure 2:**
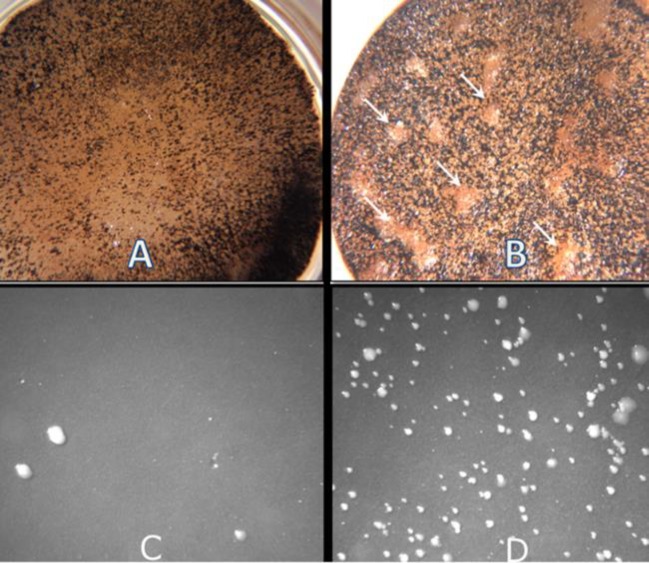
(A&B) Potato Tumor Discs Stained with Lugol’s Potassium Iodide Reagent. (A) Positive inhibitory treated potato disc showing no tumors. (B) Positive control disc; *Agrobacterium-*induced tumors appear as raised light-colored areas against a dark background. (C&D) Agar Plates with Colonies of *Salmonella typhimurium* in Ames *Salmonella*/microsome Mutagenic Test. (C) Negative Control with few colonies/plate. (D) Positive control with numerous colonies/plate. Magnification 15 X

Colonies chosen from master plates of *S. typhimurium* (strain TA100, Xenometrix, San Diego, CA) were grown in 5 ml nutrient broth #2 (Oxoid Products; Basingstoke, Hampshire, UK) at 37^o^C, to a density of 1-2 × 10^9 ^per ml (absorbance of 0.96±0.2 at 600 nm) to generate overnight cultures. Cells were diluted 1:10 immediately before using in the tests. The assay consisted of combining controls or test compounds, the *Salmonella* tester strain, the mutagen 2-aminoanthracene (2-AA) and S9 liver microsomes in a soft top agar which was poured onto a minimal agar plate lacking histidine (see Supplementary material for further details). The S9 portions had one of the following added: 10.4 μl DMSO, 10.4 μl leaf extract, 10.4 μl new growth rhizome extract or 10.4 μl mature rhizome extract. One aliquot of the duplicate was treated with 6 μl (1 mg/ml) of the mutagen 2-aminoanthracene (2-AA) and the second had 6 μl DMSO. The DMSO-treated aliquot served as a control for each treatment. All aliquots were incubated for 30 min at 37^o ^C. After 48 hr, revertant colonies on control and test plates were counted (Figure 2). Triplicate plates were made for each treatment in three separate experiments for a total of 72 plates. Data were analyzed by ANOVA (SAS Institute, Cary, NC).

For study of the mutagenic/antimutagenic activity of *Shortia* crude extracts, UV-C (254 nm) radiation exposure as the mutagen or positive control for bacterial mutation was used (see Supplementary material for further details). 

**Estrogen Receptor Binding Assay**

All chemicals were from Fisher or VWR (Atlanta, GA) or Sigma Chemical (St. Louis, MO) unless otherwise noted. Estradiol, [2,4,6,7-^3^H(N)], (71 Ci/mmol), was obtained from Perkin Elmer (Boston, MA) and human recombinant ERα and ERβ were obtained from ThermoFisher (Waltham, MA). Standards and plant extracts were prepared and assayed as reported earlier (Gray et al., 2004 ▶). Estrogen-binding equivalents (EBE) were derived from a 4-parameter logistic standard curve generated by StatLIA Analysis software (Brendan Scientific; Carlsbad, CA) where the sample concentration displaced approximately 50% ^3^H-E_2_ binding from each receptor (IC50).

**Statistical analysis**

Analysis was conducted using ANOVA on SAS for the antitumor and antimutagenic assays and StatLIA for the estrogen receptor binding assay, respectively.

## Results

**Antitumor assay**

The mean tumors/disc for the control was 7.23±3.48. The positive inhibitory treatment with camptothecin treatment resulted in 98.2% inhibition of tumors, and solvent treatment alone resulted in 0.19±1.2 tumors/disc. All concentrations of extracts significantly (p=0.05) inhibited *A. tumefaciens*-induced tumor formation on potato discs when compared to controls with no extract (Table 2). For the leaf extract, 1:10,000 dilution significantly inhibited tumor initiation when compared to 1:100,000 dilution. For all extract types, 1:100 dilutions inhibited tumor formation better than 1:100,000 dilutions. Overall, the mature rhizome extract inhibited tumor initiation better than leaf or new growth rhizome extracts (Table 3). 

**Table 1:** Chemosystematic analysis of chemicals in members of Diapensiaceae

**Chemical**	**Member**	**Reference**
**Ellagic acid**	*Shortia galacifolia*	(Harborne and Williams, 1973 ▶)
	*Berneuxia thibetica*	(Harborne and Williams, 1973 ▶)
	*Galax aphylla*	(Harborne and Williams, 1973 ▶)
**Kaempferol**	*Galax urceolata*	(Soltis et al., 1983 ▶)
**Quercetin**	*Galax urceolata*	(Soltis et al., 1983 ▶)
**Spinasterol**	*Berneuxia thibetica*	(Wang et al., 1998 ▶)

**Table 2:** Percent inhibition of tumor formation in the *Agrobacterium tumefaciens* tumor induction assay after treatment with *Shortia* crude extracts

**Sample**	**Dilution** **(Concentration)**	**Tumors/Disc** **(Mean±SD)**	**Tumor Inhibition (%)**
**Control**	NA	7.23±3.48^a*^	NA
**Leaf**	1:100 (10 mg/ml)	1.97±2.97^b^	72.6
**Leaf**	1:1,000 (1 mg/ml)	2.02±2.32^b^	72.0
**Leaf**	1:10,000 (0.1 mg/ml)	1.84±2.78^b^	74.5
**Leaf**	1:100000 (0.01 mg/ml)	2.84±2.54^c^	60.6
**New rhz**	1:100 (10 mg/ml)	1.00±1.68^b^	86.1
**New rhz**	1:1,000 (1 mg/ml)	2.42±2.69^c^	66.4
**New rhz**	1:10,000 (0.1 mg/ml)	2.40±2.58^c^	66.7
**New rhz**	1:100000 (0.01 mg/ml)	2.96±3.45^d^	59.0
**Mature rhz**	1:100 (10 mg/ml)	0.86±1.13^b^	88.0
**Mature rhz**	1:1000 (1 mg/ml)	1.37±1.85^b^	81.3
**Mature rhz**	1:10,000 (0.1 mg/ml)	1.55±2.13^b,c^	78.8
**Mature rhz**	1:100000 (0.01 mg/ml)	1.54±2.34^c^	79.2

*Notes*. Rhz=rhizome. Camptothecin control=0.14 tumors/disc; solvent control=0.19±0.72. Values with different superscripts are significantly different within sample type.

%Inhibition=[(control mean)–(test extract mean) x 100/(control mean)]

The preliminary assays, using either hexane or 80% methanol rhizome extracts of* Shortia* in the tumor induction assay, revealed that the percentage of tumor inhibition using a hexane extraction of rhizome was similar to that of methanol (data not shown). 

**Table 3:** Overall mean numbers of tumors per disc in the *Agrobacterium tumefaciens *tumor induction assay after treatment with *Shortia* crude extracts

**Treatment**	**n**	**Mean Number of Tumors Per Disc**
**Positive ** ***Agrobacterium*** ***tumefaciens*** ** Control**	60	7.23^a^
**Leaf Extract**	254	2.14^b^
**New Growth Rhizome Extract**	261	2.19^b^
**Mature Growth Rhizome** **Extract**	274	1.3^c^
**Solvent Control**	72	0.19^d^
**Camptothecin Control**	72	0.14^d^

*Note*. Numbers with different superscripts

a-d () are significantly different at p<0.05.

**Table 4:** Effect of *Shortia* leaf, new growth rhizome and mature rhizome extracts on the revertant colonies formed in Ames *Salmonella*/microsome mutagenic assay with and without addition of 2-aminoanthracene (2-AA)

**Plant Extract**	**Revertant colonies formed without 2-AA** **(Mean±SD)**	**Revertant colonies formed in presence of 2-AA** **(Mean±SD)**	**Inhibition** **(%) ** **
**Control (DMSO** **and S9)**	48.2 ± 12.8^a^*	991.6 ± 143.0^b^	NA***
**Leaf**	50.3 ± 7.30^a^	303.3 ± 112.1^c^	73.0
**New growth rhizome**	48.2 ± 6.34^a^	212.9 ± 122.3^d^	82.5
**Mature rhizome**	46.2 ± 10.9^a^	254.0 ± 100.3^c^,^d^	78.2

A=# of histidine revertants formed with 2-AA in control

B=# of histidine revertants formed with 2-AA in the presence of extract

C=# of revertants formed without 2-AA or extract in the control

* Numbers with different superscripts

a-d () are significantly different at p<0.05.

** % Inhibition = 1- (A-B) x 100/ (A-C)

*** Not applicable

**Antimutagenic assay**

Antimutagenic activity was evident in the Ames *Salmonella*/microsome assay for the crude extracts of *Shortia* leaf, new rhizome and mature growth rhizome (Table 4). All three plant extracts significantly inhibited the formation of histidine-independent revertant colonies after exposure to the mutagen 2-AA. In terms of inhibiting revertant colony formation, the new growth rhizome extract was slightly more active (p=0.03) than leaf extract, but not different from mature rhizome extract. There were no differences in the numbers of colonies formed in the DMSO control cells with no 2-AA added and the extract-treated cells with no mutagen added, indicating that the extracts expressed no mutagenic activity in the Ames *Salmonella*/microsome assay. Neither mature rhizome extract nor DMSO control-treated cells activated the 2-AA mutagenic changes in the bacterial cells without the S9 mix, suggesting that the buffers and extracts alone were incapable of activating the 2-AA or mutating the cells (Table 5). 

**Table 5:** Effect of *Shortia* mature rhizome and buffer solutions on the revertant colonies formed in Ames *Salmonella*/microsome mutagenic assay with and without addition of the S9 mix

**Treatment**	**Revertant colonies formed without 2-AA** **(Mean ± SD)**	**Revertant colonies formed in presence of 2-AA** **(Mean±SD)**
**Control (DMSO)**	57.7±2.89^a^**	756.3±121.6^b^
**Plant extract+S9 mix**	53.7±7.64^a^	389.3±75.5^c^
**Plant extract alone**	55.7±8.50^a^	59.0±14.5^a^
Buffer mix alone*	59.0±10.6^a^	56.7±9.87^a^

** Numbers with different superscripts

a-c () are significantly different (p<0.05)

Plant extracts had no effects on UV-C-treated *S*. *typhimurium* cells (Table 6). There were no significant differences in revertant bacterial CFUs formed from UV-C treated cells with or without plant extracts. Cells treated with plant extracts but not UV-C, produced the same numbers of revertant colonies as controls with no extract.

**Estrogen Receptor Binding Assay**

Dilutions of the sample extracts were used to obtain the concentration or estrogen binding equivalents that displaced approximately 50% of ^3^H-E_2_ from receptors. The value for EBE was determined from an E_2_ standard curve and adjusted for concentration/g of original plant material. In the assay for ERα, a greater affinity was shown for RRL than for *Shortia* which was non-detectable (see Table 7). However, ERβ displayed greater affinity for mature *Shortia* rhizome>*Shortia* leaf>*Shortia* new growth rhizome>>RRL.

**Table 6:** Effect of *Shortia* leaf, new growth rhizome and mature rhizome extracts on the revertant colonies formed in Ames *Salmonella*/microsome mutagenic assay after treatment with UV-C

**Plant Extract**	**Revertant colonies formed without UV-C** **Mean ± SD**	**Revertant colonies formed after treatment with UV-C** **Mean ± SD**	**Inhibition** **(%)**
**Control (DMSO)**	46.4 ± 11.0^a^*	104.1 ± 66.4^b^	NA***
**Leaf**	43.8 ± 12.4^a^	100.4 ± 58.5^b^	6.0
**New growth rhizome**	49.0 ± 12.9^a^	110.3 ± 76.7^b^	(+)10.7
**Mature rhizome**	45.8 ± 12.6^a^	97.3 ± 71.4^b^	11.8

(+) signifies % increase above the control

A=# of histidine revertants formed with UV-C in control

B=# of histidine revertants formed with UV-C in the presence of extract

C=# of revertants formed without UV-C or extract in the control

* Numbers with different letter superscripts

a-d () are significantly different at p<0.05.

** % Inhibition=1- (A-B) x 100/ (A-C)

*** Not applicable

**Table 7:** Estrogen binding equivalents (EBE) of plant extracts with recombinant estrogen receptor alpha and beta

**Extract**	**n**	**EBE** **(ng/g)±SD**	
		ERβ	ERα
**Red Raspberry leaf**	6	23.8±11.1	8.1±3.0
***Shortia*** ** new growth rhizome**	6	116.8±41.5	ND
***Shortia*** ** leaf**	6	126.0±39.0	ND
***Shortia *** **mature rhizome**	5	161.2±32.3	ND

* Estrogen binding equivalent (EBE)=concentration (ng) of estrogen receptor binding equivalents/gram test sample as determined from an E2 standard curve.

** Relative binding affinity (RBA)={EBE E2/EBE test compound} x 100, where E2 is assigned an arbitrary value of 100.

*** ND=not detectable

## Discussion

In modern medicine, plants are used as direct therapeutic agents, as raw materials for development of new synthetic products, and as markers for new compounds (Badal et al., 2017 ▶). Some useful plant-based pharmaceuticals have been derived from random, serendipitous screening of plants, such as the anticancer drugs Taxol from the Pacific yew tree, *Taxus brevifolia,* and camptothecin from the Chinese ornamental tree, *Camptotheca acuminata *(Badal et al., 2017 ▶; Shields, 2017 ▶). A chemosystematic approach can be used to supplement ethnobotanical knowledge and perhaps facilitate the selection of plants for screening (Badal et al., 2017 ▶; Larsson, 2007 ▶). 

Rapid bench-top assays can be useful as initial, rapid methods to screen plant extracts for bioactivity. The TiA is one such assay developed based on the inhibition of *Agrobacterium tumefaciens* tumor formation on potato discs. Although *Shortia* has limited ethnobotanical history, it shares bioactive chemicals with sister genera in both the Eastern and Western hemispheres (Table 1) that do have ethnomedicinal lineage (Hamel and Chiltoskey, 1975 ▶; Wang et al., 1998 ▶). Methanol extracts of *Shortia* leaf and rhizome showed inhibition of *Agrobacterium* tumor induction at several dilutions. A concentration of 100 μg/ml was chosen for initial screening of plant compounds for bioactivity (Boyd, 1997 ▶). At this dilution, *Shortia *leaf, new growth rhizome and mature rhizome extracts showed 74.5, 66.7 and 78.8% inhibition, respectively. The significant antitumor activity detected suggests that further investigations into the antitumor properties of *Shortia *are needed. 

The Ames *Salmonella/*microsome mutagenic assay revealed that extracts of *Shortia* (100 μg/ml) have no mutagenicity to the TA100 strain of *S.* *typhimurium. * Chemicals such as the aromatic amines and hydrocarbons are biologically inactive until metabolized via the cytochrome-based P450 systems (Friedman and Smith, 1984 ▶). *Shortia *extracts, however, showed no increase in the revertant colonies after treatment with human liver homogenate (S9 mix), indicating a lack of substrates that could be oxidized by the metabolic activation system to form mutagenic metabolites. In contrast, the extracts significantly inhibited S9-activated 2-AA bacterial mutagenesis, indicating that extracts were antimutagenic in the Ames test. Further analysis on the composition of *Shortia *may reveal which chemical(s) contributes to the antimutagenicity as the activity is different from that of kaempferol and quercetin, as these chemicals can be mutagenic for TA100, whereas ellagic acid generally remains antimutagenic (Friedman and Smith, 1984 ▶; Resende et al., 2012 ▶; Silva et al., 1997 ▶). 

A benefit of using the recombinant ER binding assays as opposed to cell proliferation assays and estrogen response element (ERE) regulated reporter gene assays, is that it abrogates the need for time-consuming maintenance of live animals or cell lines (Lackey et al., 2001 ▶). Additionally, these cellular methods may not specify which receptor isoform is expressed in the system nor take into account non-genomic or crosstalk effects of phytoestrogens in a cellular system (Lackey et al., 2001 ▶). The use of recombinant ERα and ERβ in receptor binding assays offers an inexpensive, rapid technique for screening compounds for potential estrogen receptor modulatory activity. Leaf and rhizome extracts from *S. galacifolia *displayed greater binding to ERβ than for ERα, and this behavior appears to be different than that of ellagic acid, which is present in RRL and chemotaxonomic analysis (Table 1) revealed was in Diapensiaceae (Harborne and Williams, 1973 ▶). The behavior of kaempferol, also present in Diapensiaceae, appears similar to the ER binding results exhibited by *Shortia,* and was implicated as contributing to ERβ binding activities found in red wine (Zoechling et al., 2009 ▶). However, the results from the antimutagenic assays indicate that kaempferol may not be acting exclusively, as the S9 microsomes increase the biotransformation to quercetin (Silva et al., 1997 ▶). The ER modulatory activity of *Shortia* also appears different than that of spinasterol which is present in *Berneuxia *(Jeon et al., 2005 ▶; Wang et al., 1998 ▶)*. *

In summary, based on ethnobotanical history and chemosystematic analysis, the antitumor, ER binding and antimutagenic activities of extracts of the rare plant, *S. galacifolia, *were examined. These results warrant further investigation into the nature of the potential anticancer activity of the plant and indicate that a combination of ethnopharmacological and chemosystematic approaches may aid in screening plant materials in the search and development of new medicines or treatments. 
